# Why Luhmann? Social systems theory for a sociology of suicide in the platform age

**DOI:** 10.3389/fsoc.2026.1868606

**Published:** 2026-06-02

**Authors:** Enrique Fernández-Vilas, Juan José Labora-González, Juan R. Coca

**Affiliations:** 1 Department of Sociology and Social Work, University of Valladolid, Valladolid, Spain; 2 Department of Political Science and Sociology, Universidade de Santiago de Compostela, Santiago de Compostela, Spain

**Keywords:** Luhmann, media effects, platform governance, re-entry, social systems theory, social theory, sociology of suicide, structural coupling

## Abstract

Suicide becomes a sociological object through the communications that make it observable, recordable, reportable, and governable. This Perspective draws on Luhmann’s social systems theory to analyse how suicide-related meanings are selected and stabilised across functionally differentiated subsystems. The argument treats suicide as a limit-case perturbation that is processed differently by health care, law, education, media, and platform governance. Clinical research remains indispensable for understanding vulnerability and intervention; the sociological question concerns how those realities are described, circulated, and institutionally acted upon. The paper foregrounds structural coupling as a central problem for prevention. Protective communication gains force when it is converted into interaction, institutional response, and continuity of care under capacity constraints. It concludes by outlining a research agenda focused on semantic distinctions, observability conditions, temporal windows, institutional bottlenecks, cross-system couplings, and governance as a modification of selection conditions.

## 1 Introduction: why Luhmann now for the sociology of suicide

Suicide remains one of the most unstable objects in the sociology of health. Since Durkheim, it has tested sociology’s capacity to explain how apparently individual events become socially patterned (Durkheim, 1951). It appears as clinical risk, epidemiological indicator, moral disturbance, legal-administrative case, media event, and platform object governed by recommendation and moderation. This plurality constitutes suicide as a configuration of observations whose differences across subsystems shape its governability (Luhmann, 1995). Scholarship often moves between individualising explanations organised around risk factors and prediction and moral framings organised around deviance, scandal, or cultural diagnosis. Both orientations produce insight, yet they leave a sociological gap concerning the communicative operations through which suicide becomes socially consequential. Observation, description, circulation, and institutional processing shape what suicide can mean in public, what can be said about it, which interventions become plausible, and which responses are implemented.

Niklas Luhmann’s social systems theory offers a way to stabilise this sociological object. Its central premise treats communication as the basic operation of social systems and as the medium through which society reproduces itself (Luhmann, 1995). Psychic systems, bodies, and suffering remain indispensable for understanding vulnerability, while a systems-theoretical sociology incorporates vulnerability by analysing how suffering becomes socially addressable as persons, cases, risks, responsibilities, and calls for help within system-specific operations (Luhmann, 1995). Suicide sociology gains a sharper question in this frame, since the problem becomes how society observes suicide, how those observations recur, how they circulate across functionally differentiated subsystems, and how this circulation conditions governance. This shift incorporates clinical insights while keeping the sociological object at the level of observation, description, and institutional processing.

The contemporary relevance of this programme becomes visible in the platform age, where suicide-related communication unfolds within an ecology of mass media and digital platforms. Attention is organised through ranking, recommendation, and virality, while governance is distributed across institutions, newsrooms, platforms, services, and communities. Visibility can therefore be amplified or suppressed at scale. Under these conditions, suicide-related discourse and institutional response form an operative environment that shapes future descriptions, future observability, and the conditions under which help-seeking becomes accessible. A Luhmannian perspective supplies a vocabulary for this environment, since functional differentiation, codes and programmes, second-order observation, selection and re-entry, structural coupling, and self-observation help explain why suicide produces heterogeneous meanings, why governance fragments, and why communicative environments matter (Luhmann, 1995, 2000a,b).

This perspective defines a specific sociological level of explanation concerned with how suicide becomes observable, describable, comparable, and governable through communication, and with the social conditions under which suicide-related meanings are selected, stabilised, amplified, suppressed, and coupled to institutional response. This delimitation matters because media-effects research, clinical suicidology, epidemiology, and systems-theoretical sociology operate at different analytical levels. A Luhmannian approach illuminates the organisation of communicative environments, while clinical, psychological, epidemiological, and biographical approaches explain suicidal action, vulnerability, and intervention.

The choice of Luhmann clarifies a specific theoretical contribution within a broader sociological field. Interactionism clarifies how stigma and deviance are produced in encounters and classifications, while Foucauldian and biopolitical approaches illuminate the governmental rationalities through which life, death, risk, and population become objects of regulation. Discourse theory foregrounds the production of subject positions and legitimate speech, affect theory draws attention to atmospheres, contagion, and embodied intensities, and political economy highlights ownership, extraction, and infrastructural asymmetry in platform environments. Systems theory adds a different contribution by explaining why suicide is reconstructed differently across autonomous subsystems and why prevention often fails at the points where these reconstructions must be coupled. It specifies the problem of cross-system translation, selection, and institutional operativity.

This work argues that Luhmann provides a strong foundation for studying suicide as a communicative phenomenon in sociology. The claim concerns the sociological object rather than psychological mechanism, since suicide becomes socially consequential through observation and description and becomes governable through the routines, codes, and infrastructures that regulate which descriptions recur. The paper outlines an empirically workable agenda for suicide sociology under contemporary media and platform conditions. The argument makes suicide sociologically legible as a problem of observability, recurrence, and cross-system coupling, and it proposes a research agenda organised around these dimensions.

## 2 From act to communication: suicide as perturbation, observation, and re-entry

A Luhmannian approach begins by distinguishing the biographical event from the communicative event. A suicide death or attempt ruptures biography and generates heterogeneous experiences across close networks, while society encounters this rupture through communications that make it observable, describable, and available for further description (Luhmann, 1995). The sociological object therefore lies in this communicative processing across systems, where naming, framing, responsibility attribution, risk designation, public circulation, silence, omission, and organisational routines determine which descriptions are suppressed and which become stabilised.

Suicide acquires the form of a limit-case perturbation through observation, as systems process contingency according to their internal structures and programmes (Luhmann, 1995; Luhmann, 2000b). Perturbation designates an irritation that becomes system-relevant through observation and selection. Because the event resists ordinary rationalisation, it often triggers intensified description, making selection decisive. Descriptions acquire social force when they stabilise as reusable forms across institutions, media formats, and platform contexts (Luhmann, 1995). Over time, these recurrent descriptions condition expectations by shaping what can be asked, what can be explained, and what interventions appear legitimate. The phenomenon therefore includes its own descriptions, since communications about suicide become part of the environment for future communications.

The concept of re-entry describes this dynamic. Once suicide enters communication, it can reappear in subsequent communications that reuse similar semantics. Re-entry designates the recursive reintroduction of distinctions within communication, thereby stabilising observability and structuring future selections (Luhmann, 1995). This reading is compatible with empirical literature identifying short-window patterns after high-salience exposures. The “Werther effect” tradition describes increases in suicide outcomes after widely reported cases, with early evidence linking suggestion to measurable short-term changes (Phillips, 1974) and later work emphasising heterogeneity by proximity, demographic characteristics, and media mix (Bridge et al., 2020). Research on protective communication describes conditions under which recovery narratives and explicit pathways to help make protective communicative alternatives available and are associated with reduced harmful outcomes, often discussed under the “Papageno effect” umbrella (Niederkrotenthaler et al., 2020, 2021). A Luhmannian reading interprets these patterns as evidence that media environments organise selection conditions under which certain suicide-related semantics become more available, recurrent, and institutionally actionable (Phillips, 1974; Niederkrotenthaler et al., 2020, 2021).

Second-order observation becomes methodologically central because it shifts attention from events to the observation of observations (Luhmann, 1995, 2000a). While first-order observation describes the event, second-order observation examines the descriptions through which the event becomes socially differentiated and recurrent. A further analytical step examines the conditions that organise recurrence, including institutional programmes, media selection, platform visibility, and governance routines that modulate what becomes observable. Suicide governance can then be understood as a reflexive attempt to regulate descriptions of suicide, which means that the intervention target becomes the selection environment in which suicide-related semantics circulate and re-enter. Second-order observation provides the methodological condition through which suicide becomes accessible as a sociological object, since observations operate upon prior observations and stabilise differences that organise meaning (Luhmann, 2000a).

## 3 Functional differentiation

Functional differentiation is decisive for suicide sociology because it explains why suicide has multiple social realities. Modern society consists of autopoietic subsystems that reproduce themselves through communications guided by binary codes and implemented through programmes (Luhmann, 1995). Clinical systems operate through the distinction healthy/ill, legal systems through legal/illegal, and mass media through information/non-information. Platform infrastructures occupy a different status from function systems in the strict Luhmannian sense, yet they govern visibility through operational distinctions such as permissible/non-permissible, recommendable/non-recommendable, and enforceable/non-enforceable content (Luhmann, 1995; Gillespie, 2018).

Suicide is processed by each subsystem according to its own code and programme, so the same event is reconstructed into system-specific forms. Clinical systems reconstruct suicide into risk categories, diagnoses, triage protocols, and follow-up routines, while law reconstructs it into reportable cases, files, responsibilities, and liabilities. Mass media reconstruct suicide-related events into newsworthiness and attention, thereby producing public reality through selection, repetition, and timing (Luhmann, 2000a). Digital platforms reconstruct suicide-related content into recommendation and moderation decisions that classify content as enforceable or non-enforceable, recommendable or non-recommendable, and socially acceptable or policy-violating within platform governance frameworks (Gillespie, 2018). Education and community settings reconstruct suicide-related events into postvention scripts, memorialisation norms, and relational repertoires that can buffer or intensify vulnerability.

These system-specific reconstructions stabilise expectations and authorise action, while also shaping what counts as legitimate explanation and which data become visible. In this frame, stigma and exclusion can be described as stabilised communicative selections that operate through repeated distinctions, constrain interlocution, and narrow the space of legitimate self-description. Classical interactionist work remains relevant because it clarifies how stigma degrades identities and reinforces boundaries of normality (Goffman, 1963), and how deviance emerges through social reaction and categorisation rather than through an intrinsic property of the act (Becker, 1963). Systems theory adds a further layer by showing how stigma becomes durable when programmes, routines, and public semantics reproduce it across subsystems. These selections appear in clinical forms such as “high-risk patient,” in media forms such as “tragic scandal,” in administrative forms such as “case file,” and in platform forms such as “policy-violating content,” each of which constrains what can be said and what can be done.

Functional differentiation also clarifies why governance fragments, since meaning and intervention are coordinated through distributed systems rather than through a single centre, and each subsystem acts rationally within its code while generating consequences for others. Media cycles can increase demand for services, platform referral features can couple vulnerable users to care or fail through friction and inaccessibility, legal reporting obligations can shape journalistic practices and institutional responses, and educational postvention protocols can influence local narratives and relational dynamics. The sociological task concerns structural coupling, understood as selective connectivity between operationally autonomous systems (Luhmann, 1995). Couplings describe how systems connect through selective reconstruction, and their quality determines whether protective communication becomes interaction and continuity of care, or whether demand, capacity, and meaning drift apart.

## 4 Media and platforms as selection infrastructures

Luhmann treated mass media as a functional system that produces reality through the selection and dissemination of information (Luhmann, 2000a). Contemporary media ecologies intertwine mass media with platforms that organise visibility through algorithmic ranking, recommendation, and content governance. A Luhmannian perspective is useful here because it treats media and platforms as infrastructures of observability, which condition the communications that gain prominence, the narratives that recur, and the semantic forms that become available for re-entry.

This framing supports a precise discussion of amplification and stabilisation. High-salience events and intense news cycles create conditions under which certain narrative forms become more selectable. Empirical work has highlighted associations between salient exposures and short-window changes in suicide outcomes, with heterogeneity that depends on identification gradients and media mix (Phillips, 1974; Bridge et al., 2020). At the level of communicative form, these dynamics correspond to the heightened prominence of closure narratives, method salience, monocausal framing, and aestheticisation. Protective dynamics correspond to communicative forms that increase the selectability of alternatives. Recovery narratives, explicit pathways to help, multicausal contextualisation, and harm-reduction guidance are documented as protective orientations in systematic reviews and meta-analyses (Niederkrotenthaler et al., 2020, 2021). The WHO’s updated guidance for media professionals codifies this evidence into operational recommendations aimed at reducing harmful salience and increasing the accessibility of help-seeking (World Health Organization, 2023).

Systems theory contributes two additions to this literature that matter for sociology. First, it treats “Werther” and “Papageno” as second-order semantic regimes of re-entry that condition recurrence across subsystems, rather than as properties of persons or messages in isolation (Luhmann, 1995, 2000a). This reading interprets media-effects literature as evidence that communicative environments organise selection conditions under which certain suicide-related semantics become more available, recurrent, and institutionally actionable. Second, systems theory highlights a coupling condition that determines whether protective communication stabilises. Protective communication raises expectations of recognition and help, while stabilisation depends on the system’s ability to convert those expectations into timely access and continuity of care. When services are overwhelmed, fragmented, or inaccessible, a protective semantic orientation can lose force, producing frustration, disengagement, or renewed harmful circulation. This is a systems statement about coupling between communicative selection and institutional operativity (Luhmann, 1995, 2000b), as well as a sociological language for a practical intuition that appears in prevention guidance, since media and platform interventions matter in relation to the capacity of services and relational environments to absorb induced demand.

Platforms introduce an additional governance layer. Referral triggers, downranking decisions, enforcement timing, and protective promotion become second-order selections that reshape visibility. Platform governance scholarship shows how moderation and recommendation systems act as contested infrastructures for public discourse, with partial transparency and distributed accountability (Gillespie, 2018). Critical platform studies adds that these infrastructures are shaped by corporate ownership, advertising markets, data extraction, jurisdictional constraints, moderation labour, and uneven capacities for contestation (van Dijck et al., 2018; Couldry and Mejias, 2019; Zuboff, 2019). Visibility is therefore an infrastructural and political-economic achievement, because some actors possess greater capacity to define categories, enforce thresholds, access moderation systems, mobilise attention, or challenge decisions, while others are rendered less visible, over-policed, or displaced into marginal communicative spaces.

A Luhmannian approach turns these dynamics into a researchable problem by asking when platform governance becomes system-relevant, under which thresholds it modifies observability, and how it couples into institutional response capacity. It also specifies how power enters communication through control over visibility, classification, temporality, and access to institutional response. This yields empirical questions suited to the sociology of health and keeps the analysis within the limits of sociological method by focusing on how communication is selected and re-selected, how those selections structure expectations, and how distributed governance modifies selection conditions under unequal conditions.

## 5 Implications for research design

A Luhmannian programme shifts suicide sociology from individual prediction to the observation of communicative dynamics and couplings (Luhmann, 1995). This shift becomes empirically workable when the object is defined with precision. Research can begin by mapping the distinctions through which suicide is described across subsystems, with the aim of identifying recurrent distinctions that organise observability. Risk/illness, tragedy/scandal, deviance/victimhood, responsibility/accident, and preventability/inevitability recur across institutional and media settings and stabilise as templates for communication. These distinctions can be studied through qualitative discourse analysis and computational approaches such as semantic networks or topic models, with the analysis centred on recurrence and selection rather than individual attitudes.

Empirical strategies can specify patterns of observability, recurrence, and coupling through time-sensitive designs that capture structured variation in communicative environments (Bernal et al., 2017). Operationalising selection conditions is central, because these conditions can be approached as observable features of media and platform environments, including reach and prominence proxies such as impressions, ranking, and dwell time, and content-level features such as method detail, sensationalism, and explicit help pathways. Platform governance signals can be treated as measurable interventions into visibility and circulation, foregrounding the environments that structure what becomes selectable. Operational mappings from semantics to measurable indicators and evaluation strategies oriented to temporal windows have been specified in a recent modelling framework, which can serve as a template for empirical extensions across jurisdictions (Fernández-Vilas and Coca, 2026).

A concrete illustration can clarify the empirical payoff of the framework. Consider a high-salience suicide-related media event involving a widely recognised public figure or a fictional representation that receives extensive news and platform circulation. A conventional media-effects approach would ask whether exposure is followed by measurable changes in suicidal behaviour within a defined temporal window, while a public-health approach would evaluate whether reporting followed established recommendations, including avoidance of method detail, sensationalism, and monocausal framing, and whether help-seeking information was provided. These questions remain necessary, while a systems-theoretical design adds a different set of observations concerning reconstruction, recurrence, re-entry, and coupling.

Such a design would first map how the event is reconstructed across subsystems, as health services may reconstruct it as a clinical risk signal, legal-administrative settings as a matter of responsibility, procedure, or liability, mass media as a newsworthy and affectively charged story, platforms as a moderation and recommendation problem, and educational or community settings as a postvention issue. The analysis would then trace distinctions that recur across these reconstructions, such as tragedy/scandal, risk/resilience, preventability/inevitability, personal suffering/public responsibility, and permissible/non-permissible visibility. It would also examine re-entry by asking which descriptions are repeated, transformed, or suppressed in subsequent communications, and it would analyse coupling by testing whether protective communication is converted into institutional response, including helpline contacts, referral completion, clinical follow-up, school postvention activity, or changes in platform governance.

The empirical advantage of the Luhmannian approach lies in showing how a communicative event becomes differently actionable across systems, where translations fail, and where prevention bottlenecks emerge. For example, a platform may downrank harmful content and promote referral messages while health services lack the capacity to absorb increased demand, in which case the relevant sociological finding concerns a failed structural coupling between protective visibility and institutional operativity.

Re-entry and temporal windows become central empirical targets because suicide-related communication often exhibits short-window dynamics following high-salience events. Research designs suited to these dynamics include interrupted time-series approaches, segmented regression, and distributed-lag specifications, used with caution regarding causal attribution, confounding, and temporal autocorrelation (Bernal et al., 2017). The analytic target concerns directional patterns, windows, and heterogeneity across contexts rather than universal coefficients. Such work can also evaluate changes following interventions, including the introduction of media guidelines, platform referrals, or service expansions, provided the analysis remains explicit about its inferential scope.

Structural coupling provides the next organising concept. Coupling analysis traces how communications in one subsystem trigger selections and responses in others, as news reporting can be followed by changes in helpline volume, platform referral features can be followed by service contact patterns, and school postvention routines can shape local narratives and relational buffering. Coupling analysis also reveals bottlenecks, which appear when protective semantic orientations produce demand that services cannot absorb, or when platforms provide referrals that fail to connect users to care because of friction, limited coverage, or constrained continuity. These bottlenecks create conditions under which governance interventions appear operationally correct while producing limited effects. Empirical work on couplings benefits from mixed methods because it requires combining time-series patterns with institutional process data, pathway tracing, and qualitative mapping of organisational routines.

Governance operates as a second-order modification of selection conditions that redistributes communicative possibilities across subsystems (Luhmann, 2000a, 2000b). Governance specifies conditions under which communications gain or lose selectability within distributed institutional environments, while prevention policies, media guidelines, and platform moderation routines function as reflexive attempts by the system to regulate its own communications about suicide (Luhmann, 2000a,b). The sociological evaluation question concerns whether these interventions modify selection conditions and shift the distribution of re-entry semantics while limiting iatrogenic side effects. Side effects can include intensification of stigma through surveillance, displacement of vulnerable discussions into less visible spaces, and reinforcement of exclusionary distinctions. Governance evaluation therefore requires attention to how interventions are perceived and processed across subsystems, how they couple into interaction and institutional operativity, and how they change the communicative availability of suicide-related scripts over time.

## 6 Discussion and conclusion

This agenda remains compatible with suicidology while retaining sociological specificity. Clinical research supplies evidence about risk, vulnerability, and intervention effects, while systems theory supplies a way to observe how those clinical realities become socially processed, described, and governed. The programme becomes cumulative when comparable indicators of observability, coupling, and capacity are developed across jurisdictions and media ecologies, since these indicators would allow theory-guided specification and iterative refinement.

This clarification also defines the scope of a sociological contribution to prevention. Media guidelines, platform governance, clinical pathways, and institutional protocols operate on objects reconstructed through different codes, temporalities, and capacities. Prevention should therefore be evaluated by the promotion of safer meanings and by the coupling of those meanings to accessible interaction, credible referral, and sustained institutional response (World Health Organization, 2023).

Suicide sociology can advance through an observation-based paradigm in which the phenomenon is constituted by communicative operations that organise meaning, recurrence, and governance across functionally differentiated subsystems (Luhmann, 1995, 2000a). This perspective improves empirical tractability by focusing on semantics, observability, re-entry, and coupling. It reframes prevention as a question of how protective communication is converted into interaction and continuity of care under institutional capacity constraints. A Luhmannian programme contributes a sociological foundation for studying why suicide-related communication becomes amplifiable or containable in contemporary communicative ecologies, and for designing research that remains explicit about its object, measurements, and limits.

## Data Availability

The original contributions presented in the study are included in the article/supplementary material, further inquiries can be directed to the corresponding author.
